# Dynamics of a Molecular Rotor Exhibiting Local Directional Rotational Preference within Each Enantiomer

**DOI:** 10.1021/acs.jpca.0c08476

**Published:** 2021-03-05

**Authors:** Kirill Nikitin, Yannick Ortin, Michael J. McGlinchey

**Affiliations:** School of Chemistry, University College Dublin, Belfield, Dublin 4, Ireland

## Abstract

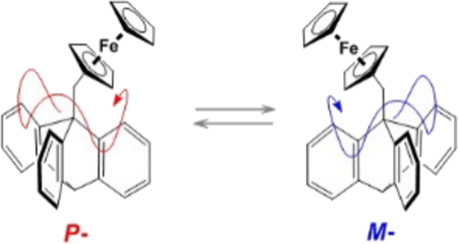

Directional internal rotation in molecular systems, generally controlled by chirality, is known to occur in natural and artificial systems driven by light or fueled chemically, but spontaneous directional molecular rotation is believed to be forbidden. We have designed a molecular rotor, whereby ferrocene and triptycene linked by a methylene bridge provide two rotational degrees of freedom. On the basis of experimental observations, in conjunction with computational data, we show that the two different modes of rotation are strongly coupled and the spatial orientation of the bistable ferrocene moiety controls the barrier to its own rotation about the triptycene axis. It is proposed that the barrier to clockwise 120° rotation across each individual triptycene blade is lower in the *M*-enantiomer and for counterclockwise 120° rotation, it is lower in its *P*-counterpart. These findings demonstrate the possibility of locally preferred thermal directional intramolecular rotation for each dynamically interconverting enantiomer.

## 1 Introduction

Directional motion is one of the key phenomena in nature. Macroscale (Newtonian) directional motion is determined by the set of initial coordinates and velocities, but on the molecular (Brownian) scale, coherent directionality is inevitably obliterated by the large number of random thermal collisions within the typical chemical event time scale.^1^ Spontaneous coherent translational motion, for a statistical molecular ensemble, is only possible due to a concentration gradient and, under equilibrium conditions, is not allowed under the Second Law.

**Figure 1:**
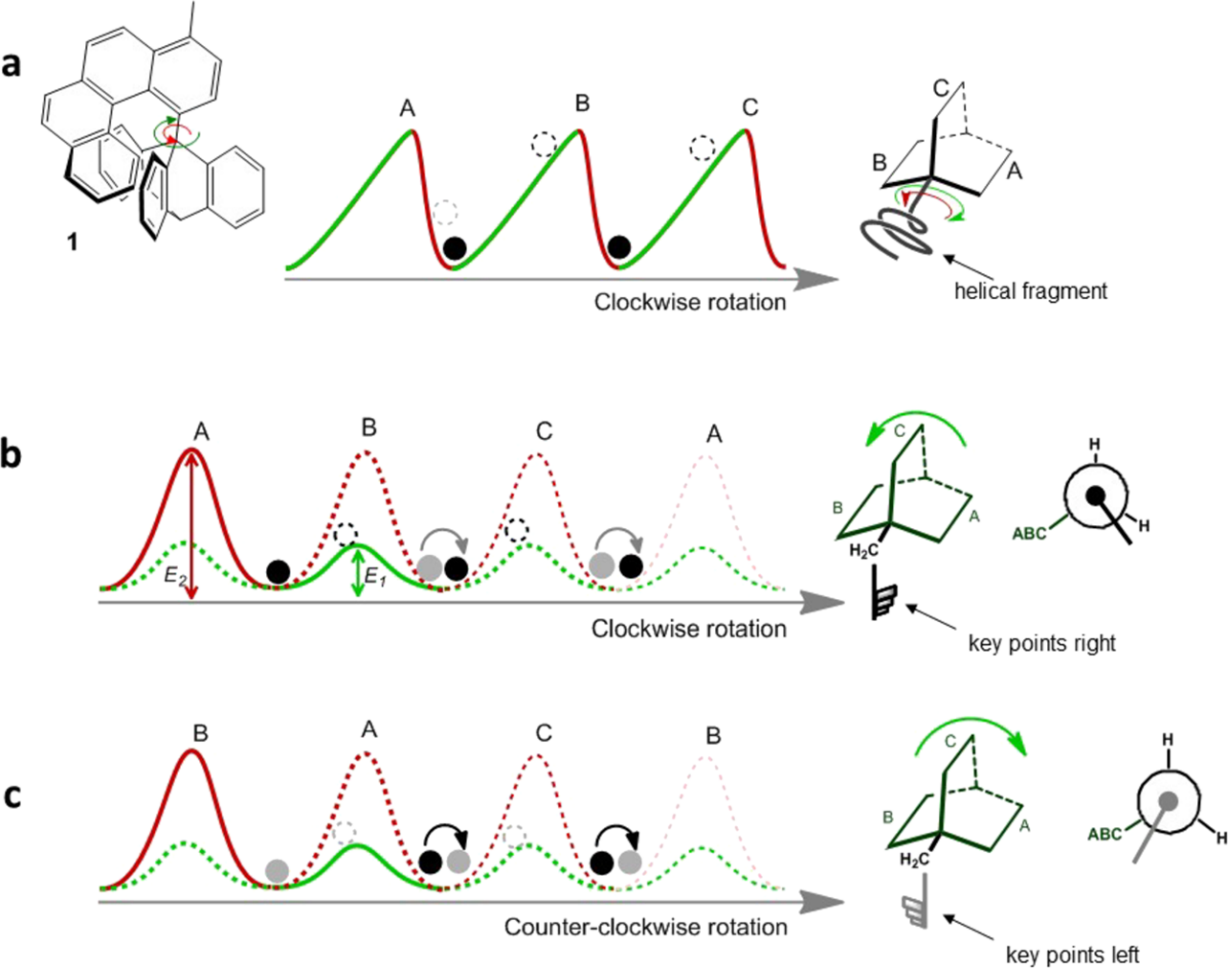
Schematics of a rotational walk in a comb-like energy profile: (a) in the unsuccessful ″ratchet″ **1**, one might, incorrectly, envisage rotation of the internally rigid helical attachment about the C–C bond proceeding preferentially clockwise by climbing the gentler gradient (green) rather than the steeper red gradient; (b) in the rotational system with two degrees of freedom, a dynamically rotatable attachment, shown as a ″key″, has two possible positions in the AB cleft: when the key points to the right (black), a counterclockwise 120° rotation of the ABC moiety proceeds easily, with low barrier *E*_1_, while the key moves smoothly over blade **B** and, due to gearing, changes its orientation; (c) in contrast, if the key is turned to the left (gray), clockwise rotation of the ABC moiety by 120° is easy and the key moves over blade **A** ending up in the **AC** cleft, but now again having the opposite sense (black) due to gearing. As the key can turn either way spontaneously, the situation repeats itself: the black key prefers the **B** → **C** → **A** sequence; gray-coded key moves in the **A** → **C** → **B** fashion.

For a single particle, diffusion in one dimension can be described by the mean square displacement as a power function of time,^2,3^ which is linear for Brownian motion. Such continuous time random walk (CTRW) formalism^4^ and waiting time distribution function^5^ can also be applied to internal molecular rotation. For molecular rotor **1** shown in Figure 1a, CTRW in a single angular dimension is analogous to a Brownian particle linear diffusion in a deep periodic potential. The rotor **1** is characterized by fixed angular jump-length (120° due to *C*_3_-symmetry of the triptycene rotor). In this work, we present a CTRW of a related molecular rotor for which our experimental data and computational results suggest local directionality.

Conceptually, the search for intramolecular rotation with a directionality preference is related to the celebrated Feynman–Smoluchowski thought-experiment ratchet with all moving parts in a single molecular system.^6^ There have been numerous approaches toward the synthesis of molecular rotors with controlled directionality,^7,8^ and the criteria to be satisfied have been analyzed from both chemical^9^ and physical^10,11^ perspectives. Elegant demonstrations of externally powered directional rotors include selective electronic excitation of a polyferrocenyl-ruthenium complex sulfur-linked to a gold surface,^12^ a chemically fueled supramolecular rotor,^13^ and a biaryl motor whereby correlated rotation led to passive controlled unidirectional motion of the rotating fragment.^14^

## 2 Methods

Molecular rotor **5** was prepared and characterized by NMR and X-ray diffractometric techniques as described elsewhere.^31^ Variable-temperature NMR spectra of **5** were acquired in dry DCM on a 500 MHz spectrometer. The experimental energy barrier was calculated based on coalescence (decoalescence) temperature and peak separation using standard techniques. The DFT-calculated molecular geometries in vacuum were optimized at the B3LYP level of theory using the 6-31G* basis set.

## 3 Results and Discussion

### 3.1 Single Degree of Intramolecular Rotational Freedom (DOF)

In the past, the quest for a power-free molecular rotation system capable of directional motion has not been fruitful. Directionality has been sought among *C*_1_-symmetric molecular rotors, such as molecule **1** (Figure 1a), having a single rotational DOF. Their rate of intramolecular rotation in each direction equals the number of intramolecular energy barrier crossing events, in a statistically uniform sample of *N* molecules, per unit time. Here, we shall denote ***J***_r_^cw^ and ***J***_r_^cc^ as the opposite rotational fluxes corresponding to clockwise and counterclockwise rotation, respectively. Hypothetically, ″directional″ operation of rotors such as molecule **1** implies that either mirror isomer, for example, the *P*-form, at equilibrium would have a nonzero net rotational flux ***J***_r_ as the vector sum of individual opposite fluxes clockwise ***J***_r_^cw^ and counterclockwise ***J***_r_^cc^ given in eq 1. Expressing vector flux ***J***_r_^cw^ and the oppositely directed ***J***_r_^cc^ (eq 2) through the unit vector ***r*** and their respective scalar rate constants *k*_r_^cw^ and *k*_r_^cc^ leads to nonequality of the two rate constants pertinent to opposite directions of rotation, as given in eq 3.
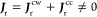
1
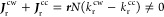
2
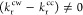
3

Therefore, eqs 1–3 set the condition of directional rotation for a single DOF molecular rotor. Of course, as the rate constants in opposite directions *k*_r_^cw^ and *k*_r_^cc^ correspond to the same energy barrier, the hypothetical directional operation of the rotor **1** in this case clashes profoundly with the microscopic reversibility principle and is forbidden.^16,17^

Figure 1a shows the energy diagram of the brilliantly designed (but ultimately unsuccessful) molecular ratchet system **1** studied by Kelly et al.,^9,18^ whereby directionality due to a different energy gradient (slope steepness) was sought. The dynamic behavior of triptycyl[4]helicene, **1**, comprising a paddlewheel-shaped *C*_3_-symmetrical triptycene moiety **ABC** and a helical molecular attachment, was monitored by elegant spin polarization transfer NMR experiments; the result was unequivocal – clockwise and counterclockwise rotations (energy barrier ∼105 kJ mol^–1^) were equally probable; in other words, ***J***_r_^cw^ + ***J***_r_^cc^ = 0. Our more recent, yet inherently analogous, chiral organometallic triptycene derivative, [η^5^-3-(9-triptycyl)indenyl]tricarbonylrhenium **2**(19) (see Figure 3a), also exhibited slow nondirectional rotation about its indenyl–triptycene linkage, albeit with a lower barrier of ∼84 kJ mol^–1^ as confirmed by 2D exchange NMR spectroscopy.

Kelly’s original observation was rationalized in an amusing and witty, but scientifically rigorous, analysis by Davis,^20^ who pointed out that ″the rates of passage across a free energy surface between isoenergetic states must be equal in both directions″; as Kelly et al. noted subsequently, ″the principle of microscopic reversibility rules″.^21,22^

### 3.2 Second Degree of Intramolecular Rotational Freedom

Introducing another rotational DOF, as depicted in Figure 1b, can, in principle, lead to an entirely different dynamic molecular function, as proposed by Alemany et al.,^23^ incorporating a rotating attachment, the ″key″, which is dynamically bistable. Being in thermal motion and turning randomly about its own axis of rotation (the second DOF), the key has a choice, at the opportune moment, to follow the lower energy pathway, barrier *E*_1_. We here present a rotor incorporating an extra DOF, such that barriers of intrinsically different heights, *E*_1_ and *E*_2_, are encountered by the rotating moiety moving in opposite directions. Since the height of the barrier depends on the direction of rotation and the angular position of the key, two different scalar rate constants, *k*_1_ and *k*_2_, are involved and the fundamental conflict with the microscopic reversibility principle is completely avoided.

As shown in Figure 1b, when the key (black, see the Newman view) is turned to the right, it encounters a high energy barrier *E*_2_ (lower rate constant *k*_2_) when traversing blade **A**, but a low energy barrier *E*_1_ (higher rate constant *k*_1_) for blade **B**, so that a 120° rotation of the **ABC** moiety is more likely to happen counterclockwise (green arrow). However, when the key is to the left (gray), as in Figure 1c, the situation is reversed and the traversal of blade **A** now faces a low barrier *E*_1_, corresponding to easy clockwise 120° rotation of the **ABC** moiety. Therefore, the criteria of directional stepwise rotation for a two-DOF molecular rotor, given in eqs 4 and 5, are
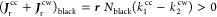
4
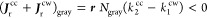
5

Of course, both situations can repeat themselves in either direction (faded dashed profiles) as the key is itself in thermal motion. Crucially, the black-coded key will tend to favor the counterclockwise 120° rotation sequence **B** → **C** → **A**, while the gray-coded key will prefer the anticlockwise **A** → **C** → **B** pathway.

**Figure 2:**
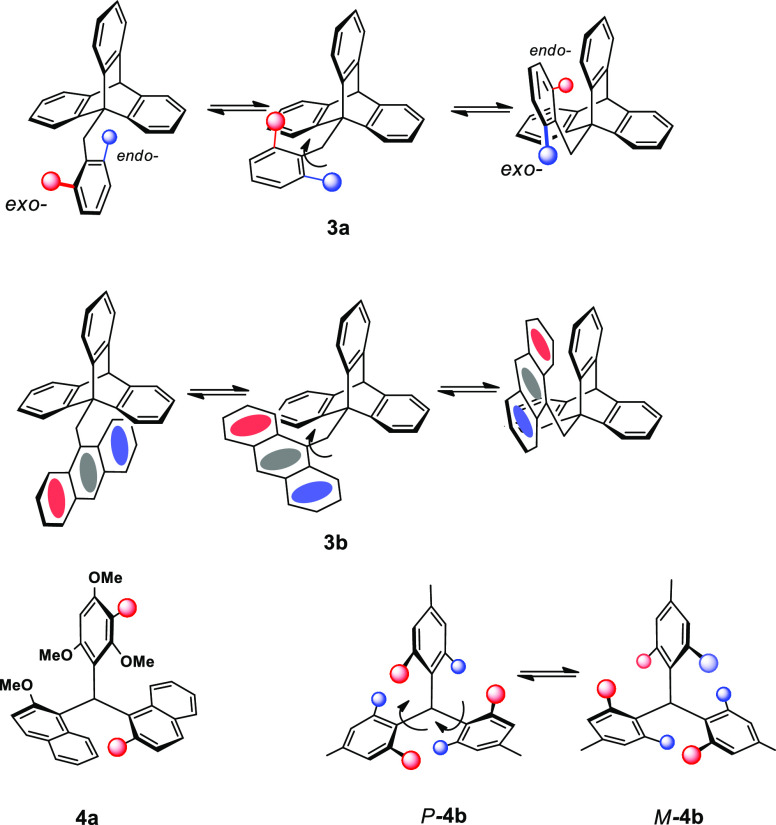
Rotational behavior of known systems (spheres are methyls): the two-DOF 9-(2,6-dimethylbenzyl)triptycene **3a** and 9-(9-anthracenylmethyl)-triptycene **3b**, each exhibiting correlated gear rotation. In the three-DOF triarylmethanes, **4a** exists as 32 slowly interconverting rotamers; inversion of the helicity in **4b** proceeds via a conrotatory two-ring flip.

In seeking experimental realization of such a situation, we consider first a well-understood example of a known system with the general formula X–CH_2_–Y, possessing two rotational DOF, about the C–X and C–Y fragments. In the ground state of 9-(2,6-dimethylbenzyl)triptycene, **3a** (Figure 2), the aryl substituent is aligned almost periplanar with the C(9)–CH_2_ linkage. As depicted in Figure 2, rotation about this bond by 120° is accompanied by rotation about the CH_2_–C(Ar) bond (the second DOF) by 180°, thus functioning as a molecular-scale 2:3 bevel gear system composed of two- and three-toothed wheels. This process was conveniently followed by monitoring the ^1^H NMR resonances of the benzyl methyls and the paddlewheel blades of the triptycene unit.^24,25^ Rotation about the three-fold axis of the triptycene equilibrated the three blades and, concomitantly, the *exo* and *endo* methyl groups. A perhaps more impressive example is provided by (9-anthracenyl)(9-triptycyl)methane, **3b**, whereby rotation about these two axes equilibrates the paddlewheel blades and also brings about interconversion of the outer benzo rings of the anthracene substituent.^24^ Most importantly, in each case, the planar character of the rotating aryl group and inherent mirror symmetry of the molecule do not differentiate clockwise or counterclockwise rotation as the respective TSs are enantiomeric, and the two opposite rotations proceed at equal rates.

However, of more direct relevance to the current situation is the remarkable in-depth study of the formidable 32-isomer system **4a** (Figure 2) by Mislow and co-workers.^26−28^ They demonstrated that all gear-like coupled rotations in it have lower energy barriers than the noncoupled rotations, leading to the presence of two sets of dynamically interconverting diastereomers. The key to the occurrence of this type of ″residual isomerism″ is the relatively rapid correlated rotation of the groups attached to the central atom. In the more symmetrical **4b**, the energetically preferred interconversion pathway linking helical *M*- and *P*-forms (barrier 91 kJ mol^–1^) occurs via a correlated movement of the bulky mesityl groups by the two-ring flip mechanism, whereby the conrotatory motion of the two flipping rings dictates the rotation of the third ring in the opposite direction, a phenomenon also observed in a Buergi and Dunitz trajectory study of the stereoisomerization pathway of triarylphosphines.^29^ In related systems, a similar behavior is found in the mono-, bis-, and tris(tricarbonylchromium) π-complexes of triphenylsilanol and the monotricarbonylchromium complex of triphenylcarbinol, in which it is the faces, rather than the edges, of the rotating aryl that are differentiated by a metal tricarbonyl attachment, an approach that can be termed “painted differently”.^30^

Considered together, these early findings appear to suggest that the preferred direction of the rotational flip can be governed by the *P*/*M* helicity. Accordingly, we aimed to explore this situation of preferred correlated rotation of two or more individual groups about single bonds in other chiral systems to investigate the possibility of energetically different diastereomeric TSs and therefore preferred local directionality when traversing them.

### 3.3 Chiral Two-DOF Molecular Rotor **5**

**Figure 3:**
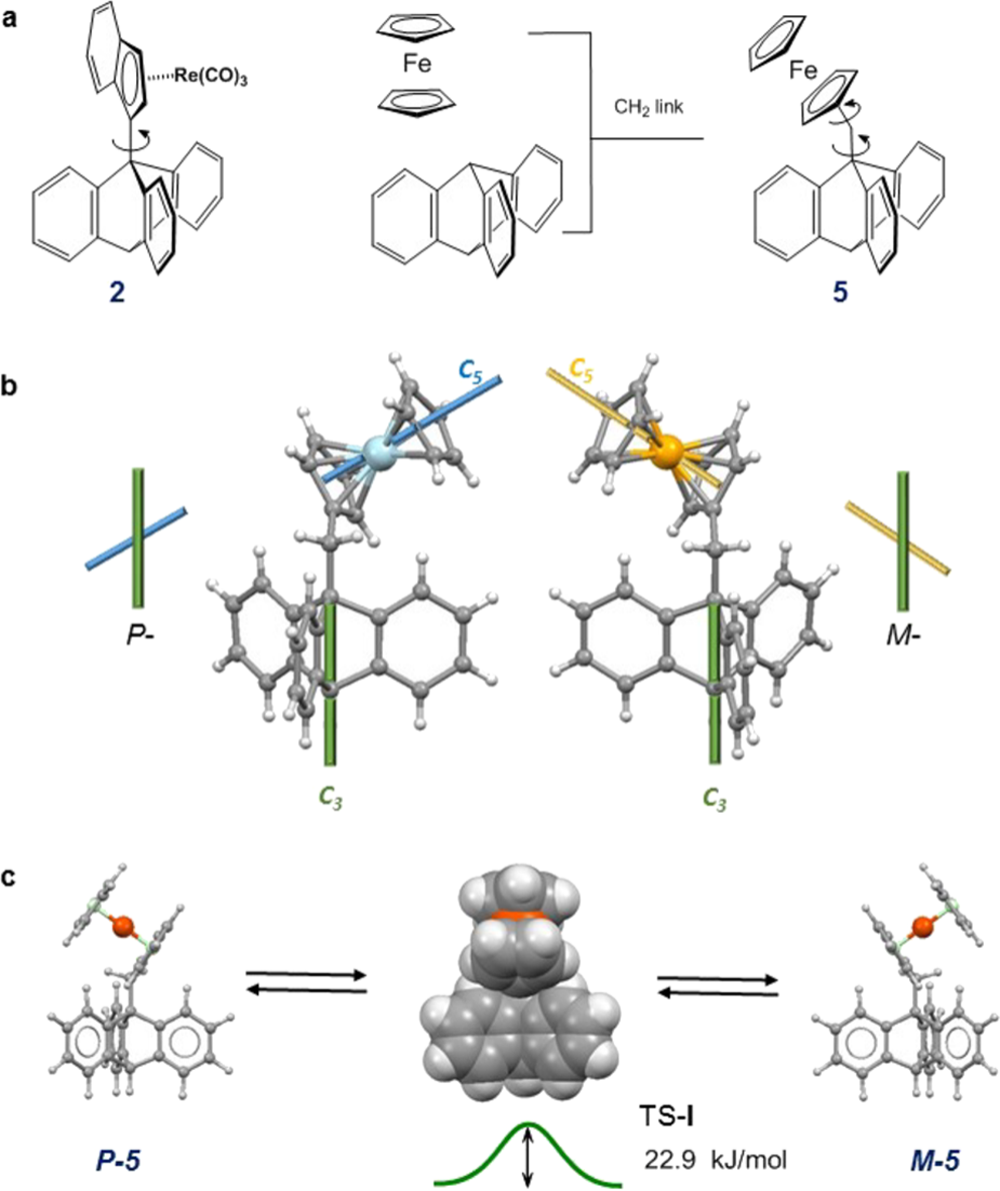
(a) Chiral rotors **2** and **5**. (b) Molecular structures of mirror antipodes *P*-**5** and *M*-**5** in the solid state: the two conformers differ in the relative helicity of nominal *C*_3_ and *C*_5_ symmetry axes. (c) In solution, the system **5** is dynamically bistable: *P***↔***M* oscillation within a valley between two blades via the low-lying mirror-symmetrical TS-**I** is very fast with a DFT-calculated energy barrier of only 22.9 kJ mol^–1^.

With the aim of extending the general idea of correlated behavior in a chiral rotor, we turned to the dynamic behavior of ferrocenyl derivatives of anthracenes and triptycenes,^15,31,32^ which is somewhat more intricate than that exhibited by mirror-symmetrical **3** and perhaps somewhat more amenable to experimental observation than the rather sophisticated **4a**. These ferrocenes can function as components of organic electroluminescent devices,^33^ combining the electrochemical behavior of the sandwich moiety with the fluorescence properties of the aromatic system.^34^ The planar anthracenyl fragment is readily converted to *C*_3*v*_-symmetric triptycyl, which reveals remarkable rotational behavior when linked to ferrocene.^15,32^ Recognizing that di-(9-triptycyl)methane and di-9-triptycyl ether exhibit dynamic gearing as a consequence of the nonlinear arrangement of the bulky triptycene rotors,^35^ we chose to introduce an angular CH_2_ linkage between a *C*_3*v*_-symmetric triptycyl rotor and a ferrocenyl group, thus providing two independent DOFs, as shown in Figure 3a. The rotor **5** was prepared by Diels–Alder addition of benzyne to 9-(ferrocenylmethyl)anthracene as we have described elsewhere.^36^ Our results suggest that in **5**, the ferrocenyl subunit plays the role of the bistable molecular ″key″ (see Figure 1b), which controls the preferred direction of rotation of the *C*_3*v*_-symmetric triptycyl moiety.

In the solid state, the new rotor **5** exists as two static noninterconverting mirror forms, *P*-**5** and *M*-**5** (Figure 3b), that differ in the relative helicity of their nominal *C*_3_ (triptycyl) and *C*_5_ (ferrocenyl) symmetry axes.^36^ Importantly, in the absence of any rotational motion (we ignore barrier-free rotation of the terminal π-cyclopentadienyl fragment of ferrocene about its *C*_5_-axis), the three triptycyl blades would be nonequivalent if probed by ^1^H or ^13^C NMR spectroscopy. The DFT-calculated *C*_1_-symmetric ground-state structures of *P*-**5** and *M*-**5** (Figure 3c) parallel closely those determined by X-ray crystallography and validate this computational approach to investigating its dynamic behavior.

### 3.4 Calculated Energy Barriers and Dynamics

Using this computational approach, we carried out calculations of detailed angular energy profiles for the rotor **5** and, having determined the energy barriers, addressed the criteria for its directional stepwise rotation. Table 1 sums up the key computational findings.

**Table 1:** DFT-Calculated Energy Profile of Molecular Rotor **5**

label	relative energy (kJ mol^–1^)	structure	imaginary frequency (cm^–1^)
5^a^	0	ground state	
TS-I^b^	22.9	oscillation barrier	i55
TS-II^b^	33.9	sliding barrier	i55
TS-III^b^	68.6	gearing barrier	i71

a Optimized to the local energy minimum.

b Optimized to the first-order saddle point.

The complex dynamics of the ferrocene unit incorporates two rotational DOFs: rotation about the triptycyl–CH_2_ bond, i.e., the molecular three-fold axis, and second, rotation about the ferrocenyl–CH_2_ linkage. The DFT calculations show that the mirror-symmetric transition state TS-**I**, shown in Figure 3c, lies only ∼23 kJ mol^–1^ above the ground state corresponding to *k*_I_ ∼10^6^ s^–1^ at ambient temperature. In practice, this fast oscillation of the ferrocene unit *within* the molecular cleft equilibrates the two triptycyl blades adjacent to the ferrocenyl unit, a process requiring a rotation of ∼115° about the Fc–CH_2_ bond together with a 20° twist of the triptycene–CH_2_ linkage (this process can be viewed in Movie S1).

**Figure 4:**
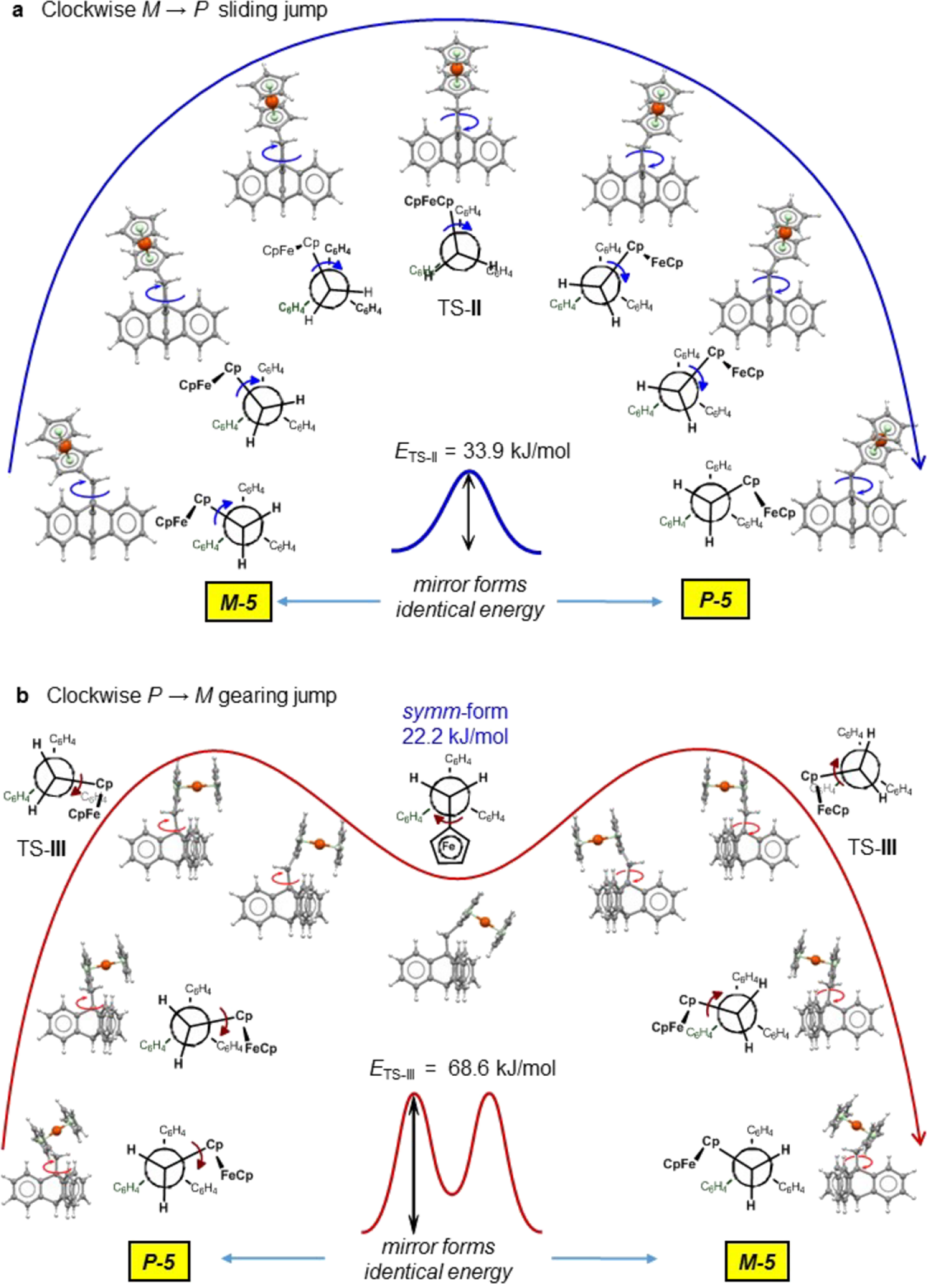
Two different calculated rotation energy profiles for the Fc unit revolving about the triptycene axis in **5**. (a) Fast sliding rotation of the ferrocenylmethyl fragment directly across a triptycyl blade in a clockwise fashion starting from *M*-**5** shown and as a series of ferrocene angular positions. The optimized TS-**II** is readily accessible energetically, 33.9 kJ mol^–1^. (b) Hindered gearing rotation in the same direction, but starting from *P*-**5**, shown as a series of interim structures including two symmetry-degenerate high-energy TS-**III** (68.6 kJ mol^–1^) linked by a local energy minimum of 22.2 kJ/mol, *symm*-**5** intermediate.

However, interconversion of the *M*- and *P*-forms of **5** can proceed not only by oscillation within a molecular cleft (Figure 3c), when no net rotation about the *C*_3_-axis of the triptycyl occurs, but also by rotation of the triptycyl–CH_2_ linkage with migration across a benzo blade, as in Figure 4. Importantly, each of the latter processes involves a concomitant ∼120° angular turn about the *C*_3_-axis of the triptycyl leading to equilibration of all three blades in **5**. In principle, each individual 120° jump can occur either by clockwise or counterclockwise 120° rotation of the Fc–CH_2_ unit, but these motions have different calculated energy barriers, *E*_1_ and *E*_2_, when starting from either *M*-**5** or *P*-**5**, because they give rise to a diastereomeric TS rather than an enantiomeric TS.

Viewed along the *C*_3_-axis clockwise sliding jump shown in Figure 4a starting from *M*-**5** can proceed with a concomitant clockwise 120° twist about the Fc–CH_2_ bond such that the Fe(C_5_H_5_) fragment maintains its *exo*-orientation in the transition state, TS-**II**, and passes upright over the adjacent benzo blade; the DFT-calculated energy of this sliding process is 33.9 kJ mol^–1^ (see Movie S2).

In contrast, a 240° clockwise two-stage gearing migration starting from *P*-**5** forces the ferrocenyl unit to pass almost “horizontally” across the triptycene blade (Figure 4b). Due to steric gearing interactions between the C_5_H_4_ ring and the proximal *ortho* proton of a benzo blade, the system has to overcome a much higher energy barrier (TS-**III**, 68.6 kJ mol^–1^) before assuming the mirror-symmetrical structure, *symm*-**5** (22.2 kJ mol^–1^ above the ground state) in which the ferrocenyl group adopts the *endo* orientation, situated in a valley between two blades, illustrated in the space-filling inset (see Movie S3).

Of course, to reach the ground state, the mirror-symmetrical *symm*-**5** would have to pass over the TS-**III** barrier once again. Such a process would parallel the correlated gear rotation mechanism previously found in 9-benzyltriptycene and its analogues **3a** and **3b**,^24,25^ but the steric requirements of a bulky three-dimensional ferrocenyl unit are considerably more demanding than those of a benzyl or anthracenyl moiety that can fit comfortably within an interblade valley.

**Figure 5:**
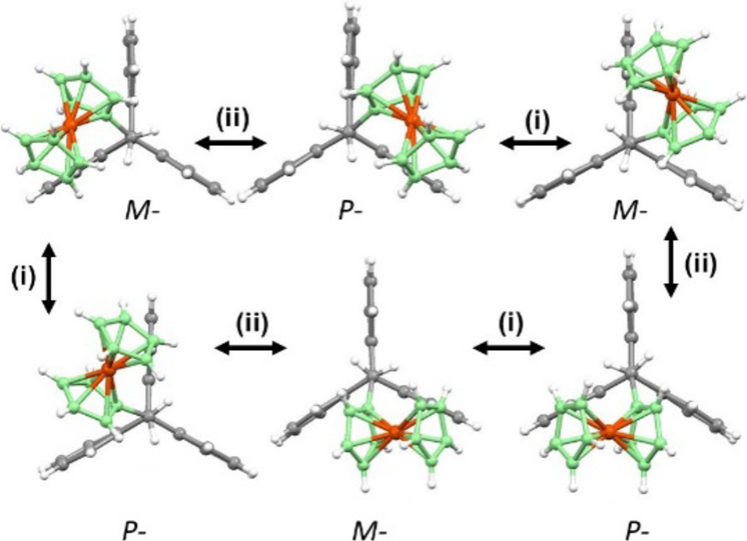
Complete rotation circuit for rotor **5** could proceed (i) via low-energy *M* ↔ *P* oscillation within a triptycene valley followed by (ii) higher-energy migration across a paddlewheel blade with inversion of the configuration.

Crucially, analysis of the favored sequence to equilibrate all three benzo blades in **5** requires the ferrocene moiety of *M*-**5** to rotate clockwise from one interblade cleft to the next (Figure 5, starting from top left, *M*-form, then (ii) and (i) successively) with concomitant inversion of chirality to become its *P*-**5** counterpart; of course, it could retrace this trajectory back to the *M*-form, but to continue its fast clockwise rotation, *P*-**5** must now undergo the low-barrier oscillation process, (i) thus regenerating its *M*-character before traversing the next barrier in the same direction. Counterclockwise rotation (Figure 5, starting from top left *M*-form, then (i) and (ii) successively) must reverse this sequence whereby the molecule first adopts the *P*-structure prior to the ferrocene moiety crossing the adjacent blade in a counterclockwise manner. As the bistable Fc–CH_2_ moiety can adopt either orientation with equal probability and rotate easily clockwise or counterclockwise, a complete 360° rotation in one direction involves three successive 120° rotations and *M*-**5** ↔ *P*-**5** oscillations, as depicted in Figure 5. Statistically, this should occur to one-eighth of all molecules in one direction and to one-eighth in the opposite direction, assuming no gear slippage.

Since each pair of scalar rotational rate constants, *k*_II_ and *k*_III_, governed by the heights of their respective energy barriers, TS-**II** and TS-**III**, are equal, i.e., (*k*_II_^cw^)*_M_* = (*k*_II_^cc^)*_P_* and (*k*_III_^cw^)*_P_* = (*k*_III_^cc^)*_M_*, the total rotational flux ***J***_r_, upon vector summation of all rotations, is zero, as given in eq 6
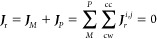
6

Nevertheless, for each mirror form, *P*-**5** and *M*-**5**, a segmentary 120° rotation rate is faster in one angular direction than in the opposite direction as given in eqs 7 and 8
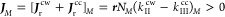
7
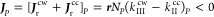
8where vectors ***J****_M_* and ***J****_P_* denote rotational flux for each interconverting mirror form individually. This outcome satisfies the directionality criteria (eqs 4 and 5) for a two DOF molecular rotor.

## 4 Experimental Observations

Undoubtedly, the low oscillation barrier between both mirror forms (Figure 3c, ∼23 kJ mol^–1^) suggests that in solution, even at low temperature, one is unlikely to see an NMR spectrum whereby all three blades are nonequivalent; however, decoalescence to see a 2:1 pattern should be accessible experimentally.

**Figure 6:**
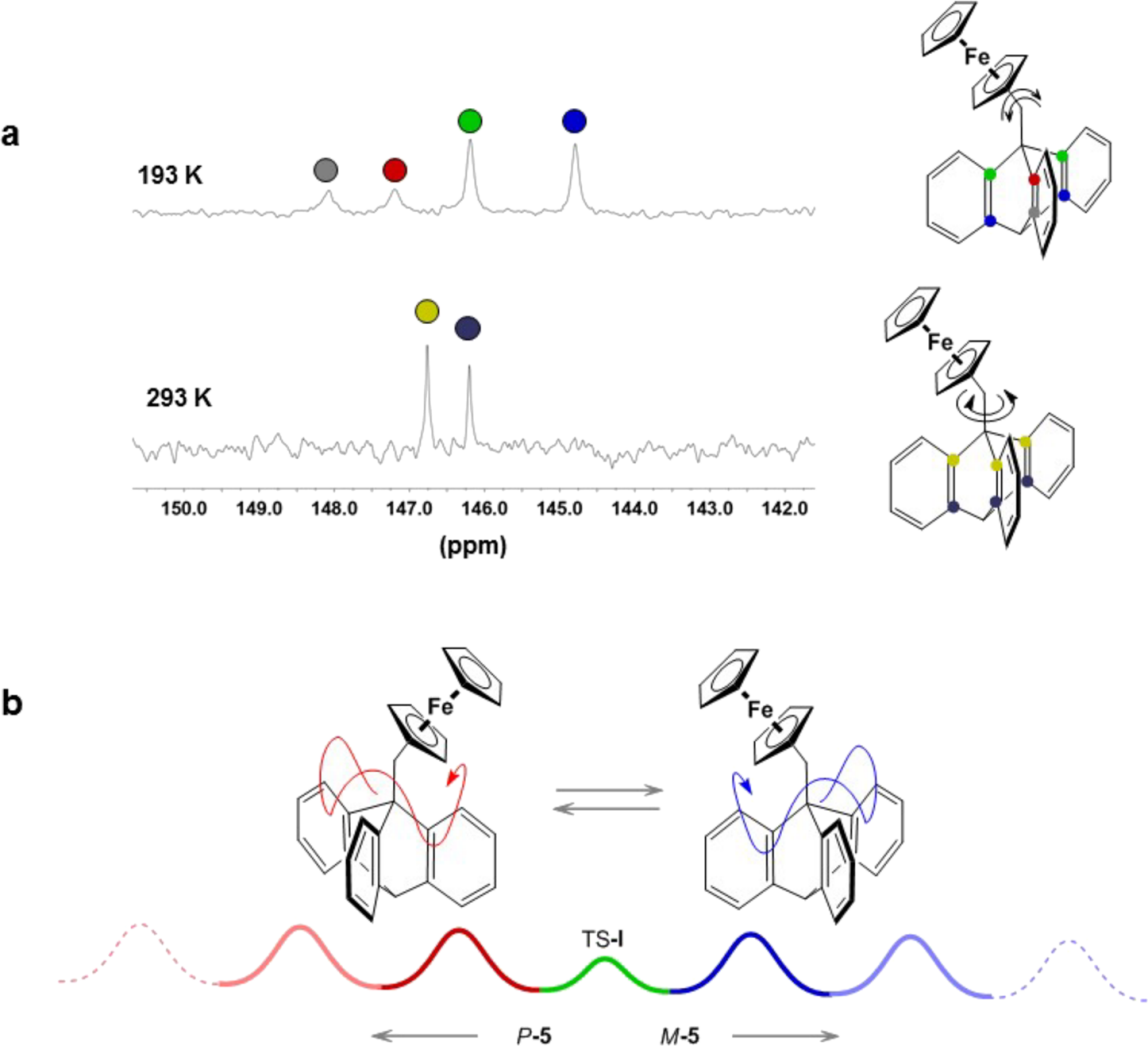
Rotational dynamics in **5**. (a) Section of the 125 MHz variable-temperature ^13^C NMR spectra (left), with the color-coded assignments of the four peaks (right). (b) In the mirror forms of **5**, ferrocenyl units undergo preferentially counterclockwise, *P*-**5** (red trajectory), and clockwise, *M*-**5** (blue trajectory) rotation. The energy diagram shows the sequence of periodically repeating rotational barriers TS-**II** in either direction. As the two processes are linked via the low oscillation barrier TS-**I**, each molecule **5** undergoes rapid changes of the preferred direction of rotation and no coherent rotation in only one direction occurs in any of the molecules.

Figure 6a shows a section of the variable-temperature 125 MHz ^13^C NMR spectrum of rotor **5** in solution, depicting the resonances for the ring junction carbons of the triptycene fragment. At ambient temperature, rotation about the ferrocenyl–methylene bond is fast on the NMR time scale, equilibrating all three triptycyl blades, and the spectrum exhibits only two equally intense peaks at 146.2 and 146.8 ppm. Gratifyingly, decoalescence is apparent at 193 K when two pairs of 2:1 resonances are observed, clearly indicating that the rotational process involving the ferrocene unit traversing the benzo blades can be slowed on the NMR time scale. An experimentally determined barrier of 41 ± 2 kJ mol^–1^ is close to the calculated energy barrier for TS-**II** (*E*_1_) and can be associated with the proposed sliding jump shown in Figure 4a. Curiously, in macroscopic terms, the rotational walk of the ferrocene unit may be described as a ″rock-and-jump″ directional ride round a carousel (Figure 6b and TOC graphics) where the ferrocenyl ″riders″ always go, for themselves, forward rather than in reverse. However, as the riders rapidly change their preferred forward orientation in thermal motion, no coherent cyclic movement of the ferrocene riders takes place, i.e., their total rotational flux is zero as given in eq 6.

## 5 Conclusions and Outlook

The two dynamically interconverting mirror-image molecular rotors, *M*-**5**/*P*-**5**, coexisting in equilibrium, individually undergo preferred directional intramolecular rotation. In essence, when there is a local (i.e., one-step) transition between two states in which two (or more) diastereomeric paths lead to a diastereomeric TS, the lower (or lowest) energy path is preferred. In this case, clockwise versus counterclockwise rotation of one rotor is favored by a coupled second rotor, thereby setting up the diastereomeric TS. A segmentary 120° clockwise sliding jump of the Fc is favored in the *M*-form, and vice versa, an entirely analogous counterclockwise jump is favored for its mirror-image *P*-form.

We do not dispute that the total internal rotational flux ***J***_r_ given by eq 6 is zero for a statistical ensemble of rotors **5**. In other words, the overall rotation rates of the ferrocene fragment about the *C*_3_-axis of triptycene are identical in both directions. However, we propose that it is favorable for each enantiomeric form to traverse *a single barrier* with a preferred direction of rotation. For each mirror form, clockwise and counterclockwise segmentary rotation rates are different, suggesting a local nonergodicity.^37^ Importantly, as both directional 120° sliding jumps are linked by a low-lying TS-**I**, the fluxional behavior of **5** is in full agreement with the principle of microscopic reversibility.^16,21,38,39^

We state very clearly that any proposal of a spontaneous thermal directionality must be made with great caution since in a contest with the Second Law, there is only going to be one winner. How then can we offer the suggestion that a directional preference for a particular mirror-form may obtain? We believe the answer is twofold. First, from a statistical point of view, energetically degenerate mirror-forms of **5** are rapidly interconverting via low-energy TS**-I** and this is a much faster process than direction-dependent segmentary rotational movement. Second, at the individual event level, unlike the situation in the helicene **1** (Figure 1a) whereby rotation occurs about a single axis and proceeds through a single barrier, in the case of **5**, rotation of the ferrocenyl unit, traversing all three benzo rings successively, one time in eight statistically, involves two rotational DOFs and, importantly, requires passage over non-isoenergetic barriers with very different diastereomeric transition-state geometries; this difference makes the energy profile for the rotation of the ferrocenyl unit about the *C*_3_-axis of the triptycene, and its rotational CTRW, direction-dependent for each individual enantiomer. We note, by comparison, that in Feringa et al.’s spectacular light-driven locked synchronous rotor, the system studied is racemic, and each individual enantiomer exhibits its own unidirectional rotation.^14^

We anticipate that, upon appropriate modification, molecular systems conceptually analogous to *M*-/*P*-**5** are well-positioned to harness classical known^12−14,40,41^ mechanisms for efficient conversion of chemical energy into directional mechanical molecular motion. The longer-term objective of obtaining an externally powered directional rotor system is very challenging. Such a systematic directional preference might involve a design where a chain of photo/electro/chemical events is triggered by an individual segmentary rotation such that the rotational energy profile would repeatedly change allowing the system, upon excitation from an external energy source, to relax preferentially in one direction.

In closing, we await with interest comments and ideas for further exegesis of the present phenomenon from both the thermodynamics and reaction dynamics perspectives.

## Supporting Information Available

Calculated molecular geometries of the critical points (PDF)
Details of preparation, characterization, NMR study, and computational study of rotor **5** (PDF)
Animation of calculated molecular oscillation movement via TS-I (MOV)
Animation of calculated molecular sliding movement via TS-II (MOV)
Animation of calculated molecular gearing movement via TS-III (MOV)

## Supplementary Material

jp0c08476_si_001.mov

jp0c08476_si_002.mov

jp0c08476_si_003.mov
