# Supplementary material for: Dynamics of a Molecular Rotor Exhibiting Local Directional Rotational Preference within Each Enantiomer
Source: J Phys Chem A. 2021 Mar 5;125(10):2061–8. doi: 10.1021/acs.jpca.0c08476 (PMC8154598; doi:10.1021/acs.jpca.0c08476)
Supplement: Supplementary file 4 — jp0c08476_si_004.pdf [file jp0c08476_si_004.pdf]

```
#
#     File Created by: Spartan '10 Export
#

@<TRIPOS>MOLECULE
M-form
58 62
SMALL
NO_CHARGES


@<TRIPOS>ATOM
   1  C1       3.297537250   -0.540903183    0.733697364    C.3   1
M-form
   2  C2       2.975663758    0.929893695    0.511374550   C.ar   1
M-form
   3  C3       2.109258704    3.553614770    0.240765856   C.ar   1
M-form
   4  C4       1.596798486    1.206475389    0.515551332   C.ar   1
M-form
   5  C5       3.909665728    1.947029846    0.356848030   C.ar   1
M-form
   6  C6       3.472618621    3.268624156    0.214977224   C.ar   1
M-form
   7  C7       1.168594470    2.526388010    0.392493218   C.ar   1
M-form
   8  H8       4.972331801    1.715183373    0.356074967      H   1
M-form
   9  H9       4.197068100    4.069638793    0.094037988      H   1
M-form
  10  H10      0.112802541    2.769988110    0.406958380      H   1
M-form
  11  C11      2.555242214   -1.319844206   -0.343596363   C.ar   1
M-form
  12  C12      0.928171729   -2.723728192   -2.105433344   C.ar   1
M-form
  13  C13      3.123275634   -2.233531755   -1.222457151   C.ar   1
M-form
  14  C14      1.170018496   -1.077582288   -0.347680124   C.ar   1
M-form
  15  C15      0.358314126   -1.799312538   -1.219209414   C.ar   1
M-form
  16  C16      2.304750703   -2.935684006   -2.115129026   C.ar   1
M-form
  17  H17      2.743406649   -3.652030432   -2.804516192      H   1
M-form
  18  H18      0.286820430   -3.277263456   -2.786288599      H   1
M-form
  19  C19      2.614335997   -0.920091548    2.046400889   C.ar   1
M-form
  20  C20      1.107126104   -1.537341365    4.298427335   C.ar   1
M-form
```

21	C21	3.237276328	-1.472830484	3.157257618	C.ar	1	M-form
22	C22	1.230426195	-0.664422818	2.047106535	C.ar	1	M-form
23	C23	0.479393742	-0.978128905	3.176132193	C.ar	1	M-form
24	C24	2.477556694	-1.782735532	4.292471445	C.ar	1	M-form
25	H25	0.514562004	-1.779907999	5.176629186	H	1	M-form
26	C26	0.712466323	-0.047970192	0.715631469	C.3	1	M-form
27	H27	4.196628656	-2.408507079	-1.206761459	H	1	M-form
28	H28	-0.715051888	-1.645847871	-1.226664058	H	1	M-form
29	H29	4.307757551	-1.664431784	3.142998194	H	1	M-form
30	H30	-0.590980299	-0.799670468	3.204275903	H	1	M-form
31	H31	-2.903907858	2.216014007	0.891446135	H	1	M-form
32	C32	-2.497238651	1.981612479	-0.085171301	C	1	M-form
33	C33	-2.886836712	2.581247536	-1.319163091	C	1	M-form
34	C34	-2.109185545	1.981866657	-2.352256811	C	1	M-form
35	H35	-2.168509979	2.205656499	-3.409531664	H	1	M-form
36	C36	-1.246499341	1.015287967	-1.754315269	C	1	M-form
37	H37	-0.530821993	0.404475432	-2.284520854	H	1	M-form
38	C38	-1.475320850	1.007473636	-0.340101163	C	1	M-form
39	Fe39	-3.220794160	0.555771557	-1.368868537	Fe	1	M-form
40	H40	-4.565749710	-0.804919329	0.719202561	H	1	M-form
41	C41	-4.499961438	-0.704834005	-0.356804154	C	1	M-form
42	C42	-3.645767806	-1.456322763	-1.218789496	C	1	M-form
43	H43	-5.962343277	0.934424556	-0.801802003	H	1	M-form
44	H44	-2.958276595	-2.233062449	-0.909714627	H	1	M-form
45	C45	-3.857892091	-1.003270411	-2.556785394	C	1	M-form
46	H46	-3.356491617	-1.372321659	-3.442188250	H	1	M-form

```
  47  C47      -4.843309466    0.028042094   -2.520119995      C  1
M-form
  48  H48      -5.216935365    0.580952773   -3.372451867      H  1
M-form
  49  C49      -5.239479189    0.213636059   -1.161542081      C  1
M-form
  50  H50      -3.645369609    3.342509929   -1.447120010      H  1
M-form
  51  H51       1.765579375    4.579804709    0.141099534      H  1
M-form
  52  H52       2.958907362   -2.216069411    5.165112712      H  1
M-form
  53  H53       4.371699897   -0.744590546    0.742111593      H  1
M-form
  54  C54      -0.808083576    0.207628438    0.766228291    C.3  1
M-form
  55  H55      -1.015825389    0.742719263    1.699769645      H  1
M-form
  56  H56      -1.295423265   -0.768802900    0.873902099      H  1
M-form
  57  Lig57    -2.043016220    1.713497655   -1.170201527     Du  1
M-form
  58  Lig58    -4.417281998   -0.584549805   -1.562808224     Du  1
M-form


@<TRIPOS>BOND
   1     2     5   ar
   2     5     6   ar
   3     3     6   ar
   4     3     7   ar
   5     4     7   ar
   6     2     4   ar
   7     6     9   1
   8     7    10   1
   9     5     8   1
  10     1     2   1
  11    11    14   ar
  12    14    15   ar
  13    12    15   ar
  14    12    16   ar
  15    13    16   ar
  16    11    13   ar
  17    16    17   1
  18    12    18   1
  19     1    11   1
  20    19    22   ar
  21    22    23   ar
  22    20    23   ar
  23    20    24   ar
  24    21    24   ar
  25    19    21   ar
```

```
   26    20    25   1
   27     1    19   1
   28    22    26   1
   29     4    26   1
   30    14    26   1
   31    13    27   1
   32    15    28   1
   33    21    29   1
   34    23    30   1
   35    34    35   1
   36    32    38   ar
   37    36    38   ar
   38    34    36   ar
   39    33    34   ar
   40    32    33   ar
   41    36    37   1
   42    40    41   1
   43    41    49   ar
   44    47    49   ar
   45    45    47   ar
   46    42    45   ar
   47    41    42   ar
   48    42    44   1
   49    45    46   1
   50    47    48   1
   51    43    49   1
   52    33    50   1
   53    31    32   1
   54     3    51   1
   55    24    52   1
   56     1    53   1
   57    54    55   1
   58    54    56   1
   59    26    54   1
   60    38    54   1
   61    39    57   1
   62    39    58   1
#
#     File Created by: Spartan '10 Export
#

@<TRIPOS>MOLECULE
TS-II
58 62
SMALL
NO_CHARGES


@<TRIPOS>ATOM
    1   C1        3.771558839    0.122240253   -0.005614274    C.3   1
TS-II
    2   C2        3.005967555    1.436369189   -0.022708716   C.ar   1
```

TS-II	3	C3	1.437884317	3.714880550	-0.050044051	C.ar	1
TS-II	4	C4	1.601917961	1.292579651	-0.017419219	C.ar	1
TS-II	5	C5	3.612088732	2.686042864	-0.041768289	C.ar	1
TS-II	6	C6	2.822886606	3.839733215	-0.055871469	C.ar	1
TS-II	7	C7	0.827008279	2.453039875	-0.030842524	C.ar	1
TS-II	8	H8	4.697196562	2.759145043	-0.045530234	H	1
TS-II	9	H9	3.289042052	4.821223711	-0.070955384	H	1
TS-II	10	H10	-0.250214046	2.408762030	-0.026817289	H	1
TS-II	11	C11	3.278332070	-0.658492756	-1.209574999	C.ar	1
TS-II	12	C12	2.129349532	-1.982517216	-3.353779486	C.ar	1
TS-II	13	C13	4.088442973	-1.149562223	-2.225909575	C.ar	1
TS-II	14	C14	1.882155577	-0.829816236	-1.230920188	C.ar	1
TS-II	15	C15	1.318026919	-1.488355299	-2.323679643	C.ar	1
TS-II	16	C16	3.510896080	-1.822713559	-3.306768339	C.ar	1
TS-II	17	H17	4.136514189	-2.210243911	-4.106165140	H	1
TS-II	18	H18	1.667804577	-2.493008918	-4.194872599	H	1
TS-II	19	C19	3.282879211	-0.624279176	1.221781444	C.ar	1
TS-II	20	C20	2.141770837	-1.886186205	3.407330673	C.ar	1
TS-II	21	C21	4.096716967	-1.086274347	2.248731725	C.ar	1
TS-II	22	C22	1.886803718	-0.794873948	1.253241626	C.ar	1
TS-II	23	C23	1.326660512	-1.421592121	2.366509437	C.ar	1
TS-II	24	C24	3.523147090	-1.728115400	3.350537613	C.ar	1
TS-II	25	H25	1.683318088	-2.372024769	4.264533971	H	1
TS-II	26	C26	1.144015068	-0.206999344	0.004030615	C.3	1
TS-II	27	H27	5.164982693	-1.003296042	-2.178580073	H	1
TS-II	28	H28	0.246484168	-1.629320924	-2.413134771	H	1

TS-II							
	29	H29	5.173073560	-0.941535154	2.193361539	H	1
TS-II							
	30	H30	0.255393706	-1.559375029	2.464007009	H	1
TS-II							
	31	H31	-1.868867570	0.814795662	2.176068529	H	1
TS-II							
	32	C32	-2.111572569	1.035959331	1.144009357	C	1
TS-II							
	33	C33	-3.109923991	1.952911871	0.696165837	C	1
TS-II							
	34	C34	-3.108798167	1.938027913	-0.731369608	C	1
TS-II							
	35	H35	-3.763959808	2.509609008	-1.376189977	H	1
TS-II							
	36	C36	-2.109796979	1.011900584	-1.158589660	C	1
TS-II							
	37	H37	-1.866162298	0.768954794	-2.185508625	H	1
TS-II							
	38	C38	-1.476263490	0.455383038	-0.000806237	C	1
TS-II							
	39	Fe39	-3.509736675	0.060995806	0.001222804	Fe	1
TS-II							
	40	H40	-3.257559481	-2.430629438	1.322516262	H	1
TS-II							
	41	C41	-3.913322235	-1.831456952	0.703830846	C	1
TS-II							
	42	C42	-3.941383396	-1.817862552	-0.723480114	C	1
TS-II							
	43	H43	-5.114587503	-0.682086870	2.209141551	H	1
TS-II							
	44	H44	-3.310407292	-2.404616381	-1.378904308	H	1
TS-II							
	45	C45	-4.942085579	-0.886723054	-1.136085723	C	1
TS-II							
	46	H46	-5.197651796	-0.637630786	-2.158069482	H	1
TS-II							
	47	C47	-5.533390556	-0.326928592	0.036109508	C	1
TS-II							
	48	H48	-6.315189218	0.421353221	0.058341137	H	1
TS-II							
	49	C49	-4.897891890	-0.909783768	1.173315051	C	1
TS-II							
	50	H50	-3.765377412	2.538528977	1.327985788	H	1
TS-II							
	51	H51	0.810471377	4.602263642	-0.060454870	H	1
TS-II							
	52	H52	4.151721818	-2.092598230	4.158413463	H	1
TS-II							
	53	H53	4.855030500	0.267304708	-0.009593926	H	1
TS-II							
	54	C54	-0.382926617	-0.583161751	0.012071699	C.3	1

```
TS-II
   55  H55       -0.565813168   -1.218179945    0.877723648      H   1
TS-II
   56  H56       -0.569010367   -1.243528343   -0.833709020      H   1
TS-II
   57  Lig57     -2.383271039    1.278836547   -0.010118062     Du   1
TS-II
   58  Lig58     -4.645614731   -1.154550984    0.010737913     Du   1
TS-II


@<TRIPOS>BOND
    1     2     5   ar
    2     5     6   ar
    3     3     6   ar
    4     3     7   ar
    5     4     7   ar
    6     2     4   ar
    7     6     9   1
    8     7    10   1
    9     5     8   1
   10     1     2   1
   11    11    14   ar
   12    14    15   ar
   13    12    15   ar
   14    12    16   ar
   15    13    16   ar
   16    11    13   ar
   17    16    17   1
   18    12    18   1
   19     1    11   1
   20    19    22   ar
   21    22    23   ar
   22    20    23   ar
   23    20    24   ar
   24    21    24   ar
   25    19    21   ar
   26    20    25   1
   27     1    19   1
   28    22    26   1
   29     4    26   1
   30    14    26   1
   31    13    27   1
   32    15    28   1
   33    21    29   1
   34    23    30   1
   35    34    35   1
   36    32    38   ar
   37    36    38   ar
   38    34    36   ar
   39    33    34   ar
   40    32    33   ar
```

```
   41    36    37   1
   42    40    41   1
   43    41    49   ar
   44    47    49   ar
   45    45    47   ar
   46    42    45   ar
   47    41    42   ar
   48    42    44   1
   49    45    46   1
   50    47    48   1
   51    43    49   1
   52    33    50   1
   53    31    32   1
   54     3    51   1
   55    24    52   1
   56     1    53   1
   57    54    55   1
   58    54    56   1
   59    26    54   1
   60    38    54   1
   61    39    57   1
   62    39    58   1
#
#     File Created by: Spartan '10 Export
#

@<TRIPOS>MOLECULE
TS-III
58 62
SMALL
NO_CHARGES


@<TRIPOS>ATOM
    1  C1       3.150691295    0.382995773    1.289287839    C.3   1
TS-III
    2  C2       2.639595777    1.774044724    1.605245099   C.ar   1
TS-III
    3  C3       1.424477922    4.191573767    2.188654711   C.ar   1
TS-III
    4  C4       1.367794607    2.036538257    1.066831941   C.ar   1
TS-III
    5  C5       3.306402901    2.703893203    2.394341697   C.ar   1
TS-III
    6  C6       2.699283746    3.929577746    2.681615588   C.ar   1
TS-III
    7  C7       0.760412893    3.251368826    1.390175338   C.ar   1
TS-III
    8  H8       4.290473040    2.468380512    2.793012953      H   1
TS-III
    9  H9       3.213751473    4.664234650    3.295277797      H   1
TS-III
```

10	H10	-0.240462702	3.497385833	1.053088762	H	1
TS-III						
11	C11	3.158078754	0.242164267	-0.222213323	C.ar	1
TS-III						
12	C12	2.873136127	-0.128020939	-2.954964010	C.ar	1
TS-III						
13	C13	4.258240037	-0.135710699	-0.982537361	C.ar	1
TS-III						
14	C14	1.901878648	0.471084496	-0.812254828	C.ar	1
TS-III						
15	C15	1.769066829	0.262325955	-2.185345890	C.ar	1
TS-III						
16	C16	4.118082797	-0.316586521	-2.362159774	C.ar	1
TS-III						
17	H17	4.972365684	-0.617538565	-2.962541171	H	1
TS-III						
18	H18	2.746823727	-0.288606249	-4.022347988	H	1
TS-III						
19	C19	2.064576842	-0.565011752	1.779302207	C.ar	1
TS-III						
20	C20	-0.032029370	-2.240199244	2.464276051	C.ar	1
TS-III						
21	C21	2.270324185	-1.582360616	2.701868605	C.ar	1
TS-III						
22	C22	0.797540866	-0.346297973	1.195534953	C.ar	1
TS-III						
23	C23	-0.235749327	-1.215335305	1.528799826	C.ar	1
TS-III						
24	C24	1.210340989	-2.421312108	3.061887858	C.ar	1
TS-III						
25	H25	-0.859904972	-2.897667986	2.718410449	H	1
TS-III						
26	C26	0.774990487	0.861448311	0.205705302	C.3	1
TS-III						
27	H27	5.218444286	-0.303583703	-0.500288489	H	1
TS-III						
28	H28	0.813588121	0.377478859	-2.685830252	H	1
TS-III						
29	H29	3.257003214	-1.724417430	3.136810345	H	1
TS-III						
30	H30	-1.191457782	-1.137474401	1.038741714	H	1
TS-III						
31	H31	-2.594563361	0.254069287	1.507105443	H	1
TS-III						
32	C32	-2.847355813	0.429870068	0.470865657	C	1
TS-III						
33	C33	-4.165279661	0.357448577	-0.070246668	C	1
TS-III						
34	C34	-4.095456217	0.697253530	-1.451794044	C	1
TS-III						
35	H35	-4.922636032	0.717677725	-2.149206691	H	1
TS-III						

```
  36  C36      -2.730365458    0.961622157   -1.758637112      C   1
TS-III
  37  H37      -2.346637932    1.254507126   -2.728666212      H   1
TS-III
  38  C38      -1.932715350    0.790678098   -0.573011431      C   1
TS-III
  39  Fe39     -2.931926711   -0.952884530   -1.086412513     Fe   1
TS-III
  40  H40      -2.387408097   -3.199004973    0.581385883      H   1
TS-III
  41  C41      -2.573167864   -2.882933877   -0.436624542      C   1
TS-III
  42  C42      -1.580942552   -2.486569355   -1.384497753      C   1
TS-III
  43  H43      -4.802472627   -3.031121647   -0.613304425      H   1
TS-III
  44  H44      -0.515167136   -2.435186843   -1.206166746      H   1
TS-III
  45  C45      -2.245380217   -2.162132703   -2.604504853      C   1
TS-III
  46  H46      -1.771856718   -1.815766898   -3.513923494      H   1
TS-III
  47  C47      -3.646566147   -2.353722286   -2.410697691      C   1
TS-III
  48  H48      -4.421267589   -2.171792128   -3.144523442      H   1
TS-III
  49  C49      -3.848924653   -2.800347646   -1.070090142      C   1
TS-III
  50  H50      -5.058208700    0.085678610    0.478041411      H   1
TS-III
  51  H51       0.930736358    5.130832049    2.423227933      H   1
TS-III
  52  H52       1.363729176   -3.213480114    3.789758791      H   1
TS-III
  53  H53       4.125711237    0.176986405    1.738853608      H   1
TS-III
  54  C54      -0.517304205    1.346153522   -0.550308409    C.3   1
TS-III
  55  H55      -0.237729784    1.434253255   -1.599667655      H   1
TS-III
  56  H56      -0.671776966    2.386573751   -0.263963253      H   1
TS-III
  57  Lig57    -3.154234500    0.647374486   -0.676564720     Du   1
TS-III
  58  Lig58    -2.778996287   -2.537141173   -1.581282996     Du   1
TS-III


@<TRIPOS>BOND
   1     2     5   ar
   2     5     6   ar
   3     3     6   ar
```

4	3	7	ar
5	4	7	ar
6	2	4	ar
7	6	9	1
8	7	10	1
9	5	8	1
10	1	2	1
11	11	14	ar
12	14	15	ar
13	12	15	ar
14	12	16	ar
15	13	16	ar
16	11	13	ar
17	16	17	1
18	12	18	1
19	1	11	1
20	19	22	ar
21	22	23	ar
22	20	23	ar
23	20	24	ar
24	21	24	ar
25	19	21	ar
26	20	25	1
27	1	19	1
28	22	26	1
29	4	26	1
30	14	26	1
31	13	27	1
32	15	28	1
33	21	29	1
34	23	30	1
35	34	35	1
36	32	38	ar
37	36	38	ar
38	34	36	ar
39	33	34	ar
40	32	33	ar
41	36	37	1
42	40	41	1
43	41	49	ar
44	47	49	ar
45	45	47	ar
46	42	45	ar
47	41	42	ar
48	42	44	1
49	45	46	1
50	47	48	1
51	43	49	1
52	33	50	1
53	31	32	1
54	3	51	1
55	24	52	1

```
   56     1     53    1
   57    54     55    1
   58    54     56    1
   59    26     54    1
   60    38     54    1
   61    39     57    1
   62    39     58    1
#
#     File Created by: Spartan '10 Export
#

@<TRIPOS>MOLECULE
Meso
58 62
SMALL
NO_CHARGES


@<TRIPOS>ATOM
    1   C1        2.982858599    1.249379364    0.000000000    C.3   1
Meso
    2   C2        2.077574272    1.316651842   -1.219788824   C.ar   1
Meso
    3   C3        0.384292769    1.121562410   -3.409749265   C.ar   1
Meso
    4   C4        1.091491096    0.312808840   -1.238762999   C.ar   1
Meso
    5   C5        2.200717930    2.224021546   -2.265403967   C.ar   1
Meso
    6   C6        1.342479565    2.130919824   -3.366285004   C.ar   1
Meso
    7   C7        0.260482853    0.212901618   -2.350602883   C.ar   1
Meso
    8   H8        2.968829187    2.993249438   -2.230468097      H   1
Meso
    9   H9        1.433361734    2.836234353   -4.187886288      H   1
Meso
   10   H10      -0.490257063   -0.561049497   -2.408575528      H   1
Meso
   11   C11       3.561560010   -0.162990670    0.000000000   C.ar   1
Meso
   12   C12       4.336934134   -2.829895569    0.000000000   C.ar   1
Meso
   13   C13       4.912821948   -0.483844297    0.000000000   C.ar   1
Meso
   14   C14       2.577588239   -1.168955264    0.000000000   C.ar   1
Meso
   15   C15       2.973264375   -2.504308001    0.000000000   C.ar   1
Meso
   16   C16       5.303544750   -1.828388146    0.000000000   C.ar   1
Meso
   17   H17       6.359059803   -2.087137322    0.000000000      H   1
```

```
Meso
   18  H18       4.635923338   -3.874846894    0.000000000     H   1
Meso
   19  C19       2.077574272    1.316651842    1.219788824   C.ar  1
Meso
   20  C20       0.384292769    1.121562410    3.409749265   C.ar  1
Meso
   21  C21       2.200717930    2.224021546    2.265403967   C.ar  1
Meso
   22  C22       1.091491096    0.312808840    1.238762999   C.ar  1
Meso
   23  C23       0.260482853    0.212901618    2.350602883   C.ar  1
Meso
   24  C24       1.342479565    2.130919824    3.366285004   C.ar  1
Meso
   25  H25      -0.273265938    1.030984874    4.270620995     H   1
Meso
   26  C26       1.116020714   -0.617649266    0.000000000    C.3  1
Meso
   27  H27       5.660804607    0.305935968    0.000000000     H   1
Meso
   28  H28       2.243534838   -3.306731670    0.000000000     H   1
Meso
   29  H29       2.968829187    2.993249438    2.230468097     H   1
Meso
   30  H30      -0.490257063   -0.561049497    2.408575528     H   1
Meso
   31  H31      -1.956742288   -1.849608704   -2.180603086     H   1
Meso
   32  C32      -2.271991621   -1.870701375   -1.145649078   C.ar  1
Meso
   33  C33      -3.608328098   -2.107481781   -0.712210530   C.ar  1
Meso
   34  C34      -3.608328098   -2.107481781    0.712210530   C.ar  1
Meso
   35  H35      -4.468658037   -2.249164504    1.353600474     H   1
Meso
   36  C36      -2.271991621   -1.870701375    1.145649078   C.ar  1
Meso
   37  H37      -1.956742288   -1.849608704    2.180603086     H   1
Meso
   38  C38      -1.417809114   -1.711547437    0.000000000   C.ar  1
Meso
   39  Fe39     -2.954017600   -0.297436081    0.000000000    Fe   1
Meso
   40  H40      -2.649892983    1.484807364   -2.181180121     H   1
Meso
   41  C41      -2.982772619    1.412878230   -1.154484511   C.ar  1
Meso
   42  C42      -2.169429254    1.614984214    0.000000000   C.ar  1
Meso
   43  H43      -5.148042153    0.859444522   -1.348471293     H   1
```

```
Meso
   44   H44      -1.118259713    1.865187794    0.000000000      H   1
Meso
   45   C45      -2.982772619    1.412878230    1.154484511   C.ar   1
Meso
   46   H46      -2.649892983    1.484807364    2.181180121      H   1
Meso
   47   C47      -4.299892674    1.082921584    0.714104535   C.ar   1
Meso
   48   H48      -5.148042153    0.859444522    1.348471293      H   1
Meso
   49   C49      -4.299892674    1.082921584   -0.714104535   C.ar   1
Meso
   50   H50      -4.468658037   -2.249164504   -1.353600474      H   1
Meso
   51   H51      -0.273265938    1.030984874   -4.270620995      H   1
Meso
   52   H52       1.433361734    2.836234353    4.187886288      H   1
Meso
   53   H53       3.755892039    2.022452680    0.000000000      H   1
Meso
   54   C54       0.108070018   -1.799698343    0.000000000    C.3   1
Meso
   55   H55       0.371488789   -2.412764747    0.869865618      H   1
Meso
   56   H56       0.371488789   -2.412764747   -0.869865618      H   1
Meso
   57   Lig57    -2.635689711   -1.933582750    0.000000000     Du   1
Meso
   58   Lig58    -3.346951968    1.321316768    0.000000000     Du   1
Meso


@<TRIPOS>BOND
    1     2     5    ar
    2     5     6    ar
    3     3     6    ar
    4     3     7    ar
    5     4     7    ar
    6     2     4    ar
    7     6     9    1
    8     7    10    1
    9     5     8    1
   10     1     2    1
   11    11    14    ar
   12    14    15    ar
   13    12    15    ar
   14    12    16    ar
   15    13    16    ar
   16    11    13    ar
   17    16    17    1
   18    12    18    1
```

```
   19      1     11    1
   20     19     22    ar
   21     22     23    ar
   22     20     23    ar
   23     20     24    ar
   24     21     24    ar
   25     19     21    ar
   26     20     25    1
   27      1     19    1
   28     22     26    1
   29     26      4    1
   30     26     14    1
   31     13     27    1
   32     15     28    1
   33     21     29    1
   34     23     30    1
   35     34     35    1
   36     32     38    ar
   37     36     38    ar
   38     34     36    ar
   39     33     34    ar
   40     32     33    ar
   41     36     37    1
   42     57     39    1
   43     40     41    1
   44     41     49    ar
   45     47     49    ar
   46     45     47    ar
   47     42     45    ar
   48     41     42    ar
   49     42     44    1
   50     45     46    1
   51     47     48    1
   52     43     49    1
   53     39     58    1
   54     33     50    1
   55     31     32    1
   56      3     51    1
   57     24     52    1
   58      1     53    1
   59     54     55    1
   60     54     56    1
   61     26     54    1
   62     54     38    1
#
#     File Created by: Spartan '10 Export
#

@<TRIPOS>MOLECULE
TS-I
58 62
SMALL
```

```
NO_CHARGES


@<TRIPOS>ATOM
    1  C1        3.271104261   -0.336914087    1.308679950    C.3   1
TS-I
    2  C2        2.836341199    1.076653125    0.972377089   C.ar   1
TS-I
    3  C3        1.981106093    3.504021553   -0.057157033   C.ar   1
TS-I
    4  C4        1.614253485    1.129889069    0.278529677   C.ar   1
TS-I
    5  C5        3.587369860    2.223000279    1.203473046   C.ar   1
TS-I
    6  C6        3.146995111    3.451973269    0.701580166   C.ar   1
TS-I
    7  C7        1.223689074    2.346443582   -0.273908097   C.ar   1
TS-I
    8  H8        4.525622904    2.156184734    1.749520029      H   1
TS-I
    9  H9        3.729368193    4.352690667    0.876289056      H   1
TS-I
   10  H10       0.347475075    2.408507660   -0.899955986      H   1
TS-I
   11  C11       3.264959648   -1.085416450   -0.028515978   C.ar   1
TS-I
   12  C12       2.979808637   -2.364263156   -2.481011216   C.ar   1
TS-I
   13  C13       4.346394405   -1.755138158   -0.582301598   C.ar   1
TS-I
   14  C14       2.022168377   -1.045942865   -0.694450949   C.ar   1
TS-I
   15  C15       1.885446319   -1.687318722   -1.920935008   C.ar   1
TS-I
   16  C16       4.203002137   -2.400134318   -1.818297828   C.ar   1
TS-I
   17  H17       5.047620337   -2.925940877   -2.255529249      H   1
TS-I
   18  H18       2.866085742   -2.863841713   -3.439505950      H   1
TS-I
   19  C19       2.159948485   -0.972427093    2.122294332   C.ar   1
TS-I
   20  C20       0.045650334   -2.381640675    3.231081515   C.ar   1
TS-I
   21  C21       2.323975269   -1.603179912    3.349906753   C.ar   1
TS-I
   22  C22       0.921142903   -0.978174277    1.456851990   C.ar   1
TS-I
   23  C23      -0.114564812   -1.732206142    2.000946490   C.ar   1
TS-I
   24  C24       1.252046162   -2.297297140    3.920376134   C.ar   1
TS-I
```

25	H25	-0.779445933	-2.958279833	3.640837683	H	1	TS-I
26	C26	0.930845404	-0.251074644	0.084747066	C.3	1	TS-I
27	H27	5.299066219	-1.777779660	-0.057703581	H	1	TS-I
28	H28	0.940591895	-1.673286323	-2.456165300	H	1	TS-I
29	H29	3.290222914	-1.577817960	3.848715512	H	1	TS-I
30	H30	-1.045042454	-1.850374472	1.468379891	H	1	TS-I
31	H31	-2.719867848	-1.070635454	1.131604815	H	1	TS-I
32	C32	-2.646079379	-0.046208329	0.799590472	C	1	TS-I
33	C33	-3.590937853	0.968328344	1.131744329	C	1	TS-I
34	C34	-3.201126511	2.160732805	0.459781224	C	1	TS-I
35	H35	-3.716016242	3.112129933	0.487823169	H	1	TS-I
36	C36	-2.019614965	1.872344239	-0.283312730	C	1	TS-I
37	H37	-1.522759689	2.581438123	-0.927979958	H	1	TS-I
38	C38	-1.636444709	0.504271440	-0.063262482	C	1	TS-I
39	Fe39	-3.543861558	0.657701200	-0.894525737	Fe	1	TS-I
40	H40	-3.567552151	-1.873808079	-2.171594318	H	1	TS-I
41	C41	-4.005086126	-0.883493372	-2.182616753	C	1	TS-I
42	C42	-3.515594287	0.242701074	-2.912074117	C	1	TS-I
43	H43	-5.765673589	-1.092384486	-0.809480850	H	1	TS-I
44	H44	-2.642173115	0.255011020	-3.551730826	H	1	TS-I
45	C45	-4.377591099	1.349095590	-2.646485515	C	1	TS-I
46	H46	-4.271813515	2.349904158	-3.045071157	H	1	TS-I
47	C47	-5.398287525	0.907548947	-1.752100156	C	1	TS-I
48	H48	-6.195655766	1.517229101	-1.346451287	H	1	TS-I
49	C49	-5.169346847	-0.472158625	-1.466484467	C	1	TS-I
50	H50	-4.459016718	0.841734425	1.765616813	H	1	TS-I

```
  51  H51       1.652983393    4.444563993   -0.491971489      H  1
TS-I
  52  H52       1.372962605   -2.794337621    4.879122611      H  1
TS-I
  53  H53       4.241905619   -0.375304413    1.809901714      H  1
TS-I
  54  C54      -0.436708387   -0.222466402   -0.665932672    C.3  1
TS-I
  55  H55      -0.724490720   -1.265156522   -0.844158972      H  1
TS-I
  56  H56      -0.245400103    0.200303185   -1.659100270      H  1
TS-I
  57  Lig57    -2.618840683    1.091893700    0.408908163     Du  1
TS-I
  58  Lig58    -4.493181177    0.228738723   -2.191952201     Du  1
TS-I


@<TRIPOS>BOND
   1     2     5   ar
   2     5     6   ar
   3     3     6   ar
   4     3     7   ar
   5     4     7   ar
   6     2     4   ar
   7     6     9   1
   8     7    10   1
   9     5     8   1
  10     1     2   1
  11    11    14   ar
  12    14    15   ar
  13    12    15   ar
  14    12    16   ar
  15    13    16   ar
  16    11    13   ar
  17    16    17   1
  18    12    18   1
  19     1    11   1
  20    19    22   ar
  21    22    23   ar
  22    20    23   ar
  23    20    24   ar
  24    21    24   ar
  25    19    21   ar
  26    20    25   1
  27     1    19   1
  28    22    26   1
  29     4    26   1
  30    14    26   1
  31    13    27   1
  32    15    28   1
  33    21    29   1
```

```
   34    23    30    1
   35    34    35    1
   36    32    38    ar
   37    36    38    ar
   38    34    36    ar
   39    33    34    ar
   40    32    33    ar
   41    36    37    1
   42    40    41    1
   43    41    49    ar
   44    47    49    ar
   45    45    47    ar
   46    42    45    ar
   47    41    42    ar
   48    42    44    1
   49    45    46    1
   50    47    48    1
   51    43    49    1
   52    33    50    1
   53    31    32    1
   54     3    51    1
   55    24    52    1
   56     1    53    1
   57    54    55    1
   58    54    56    1
   59    26    54    1
   60    38    54    1
   61    39    57    1
   62    39    58    1
#
#     File Created by: Spartan '10 Export
#

@<TRIPOS>MOLECULE
P-form
58 62
SMALL
NO_CHARGES


@<TRIPOS>ATOM
    1   C1        3.510238280   -0.154673059    0.828597222    C.3   1
P-form
    2   C2        2.972635120    1.267568932    0.755793642   C.ar   1
P-form
    3   C3        1.811411270    3.762543531    0.383935892   C.ar   1
P-form
    4   C4        1.725444790    1.361232881    0.112578203   C.ar   1
P-form
    5   C5        3.624439370    2.402863476    1.221751897   C.ar   1
P-form
    6   C6        3.036301266    3.659304931    1.039325643   C.ar   1
```

P-form
7 C7 1.153912498 2.616806581 -0.082872837 C.ar 1
P-form
8 H8 4.588610442 2.313720298 1.717241735 H 1
P-form
9 H9 3.539764776 4.551447554 1.402337127 H 1
P-form
10 H10 0.200919988 2.719184717 -0.588276175 H 1
P-form
11 C11 3.541565056 -0.658566479 -0.612513920 C.ar 1
P-form
12 C12 3.291257674 -1.540554678 -3.236203853 C.ar 1
P-form
13 C13 4.653896216 -1.157557605 -1.276764342 C.ar 1
P-form
14 C14 2.289981819 -0.587298522 -1.252438011 C.ar 1
P-form
15 C15 2.169128033 -1.035070921 -2.564673800 C.ar 1
P-form
16 C16 4.527809347 -1.600690243 -2.599466337 C.ar 1
P-form
17 H17 5.394385689 -1.993474320 -3.124560881 H 1
P-form
18 H18 3.189164268 -1.887347305 -4.261139824 H 1
P-form
19 C19 2.444560129 -0.992760116 1.522134836 C.ar 1
P-form
20 C20 0.350841725 -2.547471964 2.481211727 C.ar 1
P-form
21 C21 2.644083569 -1.801460482 2.634146819 C.ar 1
P-form
22 C22 1.191283127 -0.932243010 0.887546677 C.ar 1
P-form
23 C23 0.151526349 -1.727990286 1.361783564 C.ar 1
P-form
24 C24 1.587531170 -2.579972170 3.121283834 C.ar 1
P-form
25 H25 -0.467885576 -3.161423646 2.847445007 H 1
P-form
26 C26 1.162892817 -0.004383522 -0.353112114 C.3 1
P-form
27 H27 5.615317308 -1.205993389 -0.770667347 H 1
P-form
28 H28 1.214955206 -1.004851560 -3.081076522 H 1
P-form
29 H29 3.619428448 -1.836433469 3.114140360 H 1
P-form
30 H30 -0.819276291 -1.711311184 0.879034664 H 1
P-form
31 H31 -1.284833771 0.432134072 1.744950645 H 1
P-form
32 C32 -1.758356349 0.906893764 0.898249297 C.ar 1

```
P-form
  33  C33      -2.885186719    1.779228790    0.970099311   C.ar   1
P-form
  34  C34      -3.200489054    2.206294219   -0.352953060   C.ar   1
P-form
  35  H35      -4.014696510    2.860676003   -0.635863085     H   1
P-form
  36  C36      -2.261868247    1.595988182   -1.236814211   C.ar   1
P-form
  37  H37      -2.233351530    1.718322014   -2.312932485     H   1
P-form
  38  C38      -1.356404178    0.787839005   -0.471851810   C.ar   1
P-form
  39  Fe39     -3.324359705    0.162798574   -0.220643949    Fe   1
P-form
  40  H40      -4.087472057   -1.661734842    1.805425801     H   1
P-form
  41  C41      -4.229505665   -1.405722849    0.763650693   C.ar   1
P-form
  42  C42      -3.455204856   -1.892781207   -0.333560207   C.ar   1
P-form
  43  H43      -5.945220729    0.037606846    0.824337719     H   1
P-form
  44  H44      -2.632359938   -2.593916285   -0.269071166     H   1
P-form
  45  C45      -3.960760473   -1.297138892   -1.528201020   C.ar   1
P-form
  46  H46      -3.585809245   -1.456304476   -2.531060733     H   1
P-form
  47  C47      -5.046633514   -0.442369163   -1.170393531   C.ar   1
P-form
  48  H48      -5.627795743    0.161380261   -1.855511796     H   1
P-form
  49  C49      -5.212567889   -0.510165560    0.245782714   C.ar   1
P-form
  50  H50      -3.415333292    2.054200144    1.872937861     H   1
P-form
  51  H51       1.355777088    4.737378642    0.231983040     H   1
P-form
  52  H52       1.737274381   -3.215416761    3.990036954     H   1
P-form
  53  H53       4.484551742   -0.215258879    1.320900382     H   1
P-form
  54  C54      -0.187027149    0.046656386   -1.097529441    C.3   1
P-form
  55  H55      -0.470491499   -0.987733724   -1.324220141     H   1
P-form
  56  H56      -0.005846782    0.520827194   -2.069156826     H   1
P-form
  57  Lig57    -2.292460909    1.455248792   -0.038654094    Du   1
P-form
  58  Lig58    -4.380934479   -1.109635534   -0.404544270    Du   1
```

P-form

```
@<TRIPOS>BOND
    1     2     5   ar
    2     5     6   ar
    3     3     6   ar
    4     3     7   ar
    5     4     7   ar
    6     2     4   ar
    7     6     9   1
    8     7    10   1
    9     5     8   1
   10     1     2   1
   11    11    14   ar
   12    14    15   ar
   13    12    15   ar
   14    12    16   ar
   15    13    16   ar
   16    11    13   ar
   17    16    17   1
   18    12    18   1
   19     1    11   1
   20    19    22   ar
   21    22    23   ar
   22    20    23   ar
   23    20    24   ar
   24    21    24   ar
   25    19    21   ar
   26    20    25   1
   27     1    19   1
   28    22    26   1
   29    26     4   1
   30    26    14   1
   31    13    27   1
   32    15    28   1
   33    21    29   1
   34    23    30   1
   35    34    35   1
   36    32    38   ar
   37    36    38   ar
   38    34    36   ar
   39    33    34   ar
   40    32    33   ar
   41    36    37   1
   42    57    39   1
   43    40    41   1
   44    41    49   ar
   45    47    49   ar
   46    45    47   ar
   47    42    45   ar
   48    41    42   ar
```

49	42	44	1
50	45	46	1
51	47	48	1
52	43	49	1
53	39	58	1
54	33	50	1
55	31	32	1
56	3	51	1
57	24	52	1
58	1	53	1
59	54	55	1
60	54	56	1
61	26	54	1
62	54	38	1
