# Supplementary material for: Dynamics of a Molecular Rotor Exhibiting Local Directional Rotational Preference within Each Enantiomer
Source: J Phys Chem A. 2021 Mar 5;125(10):2061–8. doi: 10.1021/acs.jpca.0c08476 (PMC8154598; doi:10.1021/acs.jpca.0c08476)
Supplement: Supplementary file 5 — jp0c08476_si_005.pdf [file jp0c08476_si_005.pdf]

Supplementary Materials for

Dynamics of a Molecular Rotor Exhibiting Local Directional Rotational Preference within Each Enantiomer

Kirill Nikitin, Yannick Ortin, Michael J. McGlinchey

School of Chemistry, University College Dublin, Belfield, Dublin 4, Ireland

CONTENTS:

Molecular rotor **5**

Quantum Chemistry

Table S1

Figures S2 and S3

Movies S1 - S3

Molecular rotor 5

This compound was prepared as described elsewhere (ref 15 main text). Data base file CCDC 1583182 contains the X-ray crystallographic data for this structure.

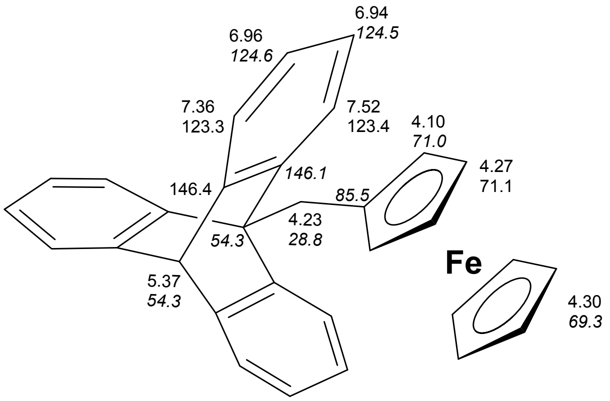

^{1}H NMR (400 MHz, 298 K, $CDCl_3$) δ = 7.52 (3H, d, *J* = 8 Hz), 7.36 (3H, d, *J* = 8 Hz), 6.96 (3H, m), 6.94 (3H, m), 5.37 (1H, s), 4.23 (s, 2H,), 4.30 (5H, s), 4.27 (2H, t, *J* = 1.8 Hz), 4.10 (2H, t, *J* = 1.8 Hz); ^{13}C NMR (100 MHz): δ = 146.4, 146.1, 124.6, 124.5, 123.4, 123.3, 85.5, 71.1, 71.0, 69.3, 54.3, 28.8.

^{1}H NMR (400 MHz, 273 K, CD_2Cl_2) δ = 7.54 (3H, d, *J* = 8 Hz), 7.40 (3H, d, *J* = 8 Hz), 7.02 (3H, m), 7.00 (3H, m), 5.42 (1H, s), 4.34 (5H, s), 4.28 (2H, t, *J* = 1.8 Hz), 4.26 (s, 2H,), 4.14 (2H, t, *J* = 1.8 Hz); ^{13}C NMR (100 MHz): δ = 146.4, 146.1, 124.6, 124.5, 123.4, 123.3, 85.5, 71.1, 71.0, 69.3, 54.3, 28.8.

Variable-temperature NMR spectra of **5** were acquired in dry DCM on an Agilent VNMRS 500 MHz spectrometer and are displayed in Figure S1. Energy barriers were calculated based on experimentally observed coalescence (decoalescence) temperature and peak separation using standard techniques (Sandstrom, J. Dynamic NMR Spectroscopy, Academic Press: London, UK; New York, NY, USA, 1982).

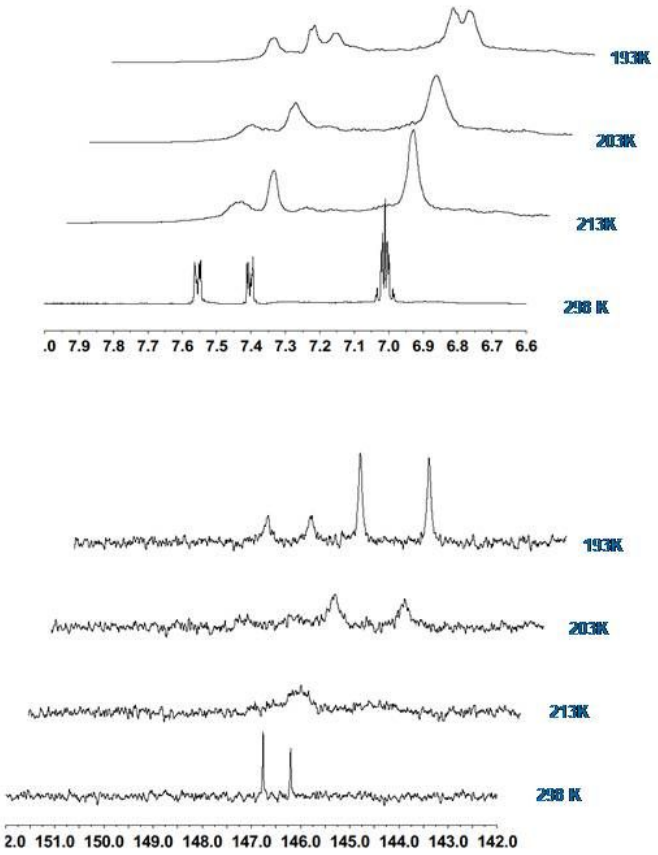

Figure S1. Variable temperature NMR spectra (chemical shift, ppm) of rotor **5** in in CD_2Cl_2.

Top: In the ^{1}H spectra of **5**, decoalescence of signals at 7.03 ppm is observed at 203K; this corresponds to a rotational energy barrier of 42.1 kJ/mol.

Bottom: In the ^{13}C spectra of **5**, decoalescence of signals at 146. 2 and 146.8 ppm is observed at 213K; this corresponds to a rotational energy barrier of 40.0 kJ/mol.

Quantum Chemistry

Quantum Chemistry calculations were carried out using Spartan10 suite of software version 10.1.0. Critical point molecular geometries in vacuum were optimized at DFT B3LYP level of theory with the 6-31G* basis set (default convergence criterion 0.0004 Ht/bohr).

Shown in Figure S3 are calculated molecular structures of the six critical points of **5**. For comparison, shown in Figure S4 are photographs of metal stick model of the rotor **5**.

Calculated total and relative energies of six critical points of molecule **5** are given in Table S1. This table also includes Imaginary vibrational frequencies for all transition state structures.

Supplementary data file "computed_structures.mol2" contains sets of atom coordinate data for each structure.

Table S1.

DFT B3LYP/6-31G* calculated critical points on the energy profile of molecular rotor **5** (vacuum).

Label	Rel. energy, kJ/mol	Total energy, Ht	Comment	Imaginary frequency, cm^{-1}
P-**5**	0	-2459.37634	energy min	-
TS-**I**	22.9	-2459.3676	oscillation TS	i55
M-**5**	0.2	-2459.37625	energy min	-
TS-**II**	33.9	-2459.36343	sliding TS	i55
symm-**5**	22.2	-2459.3679	local energy min	-
TS-**III**	68.6	-2459.35023	gearing TS	i71

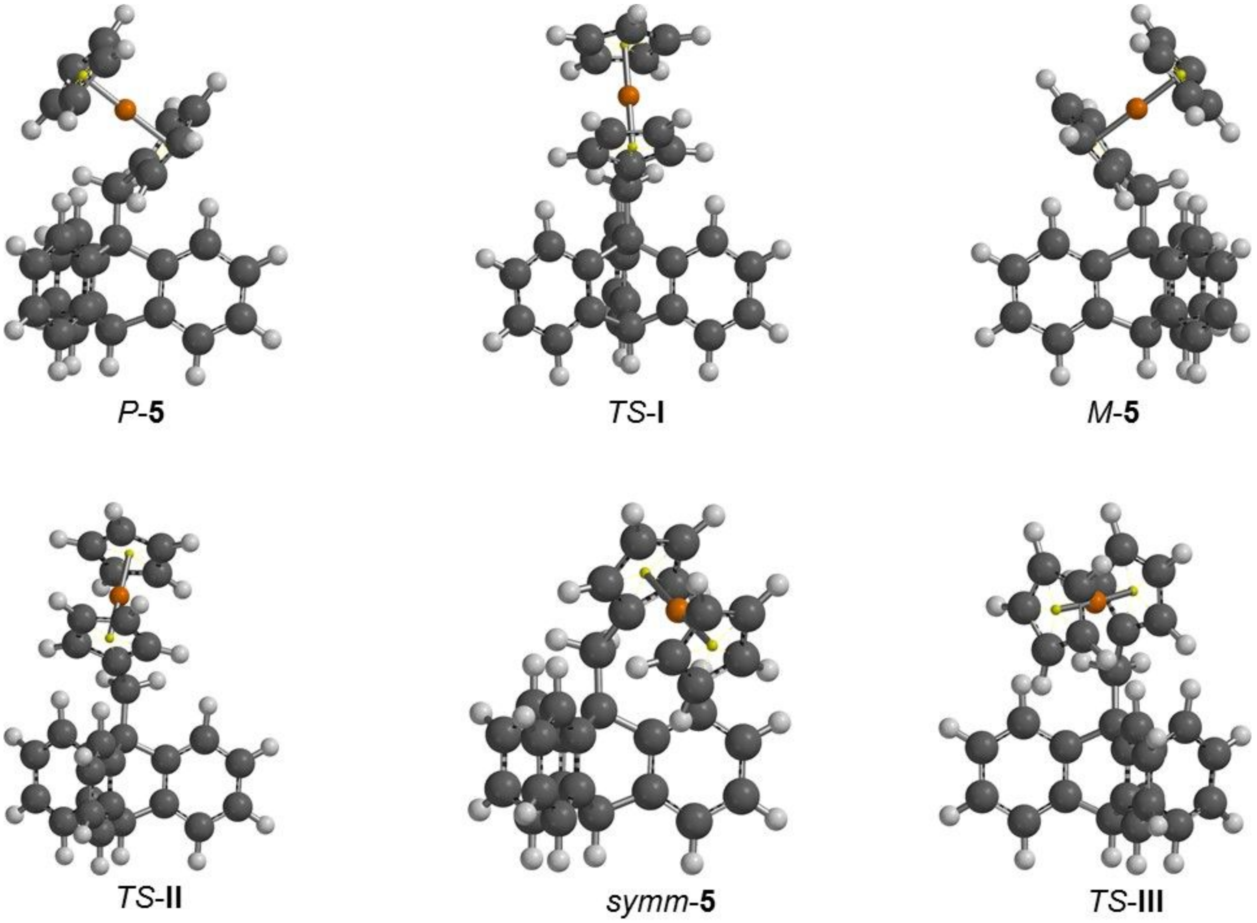

Figure S2. DFT calculated critical points of rotor **5.**

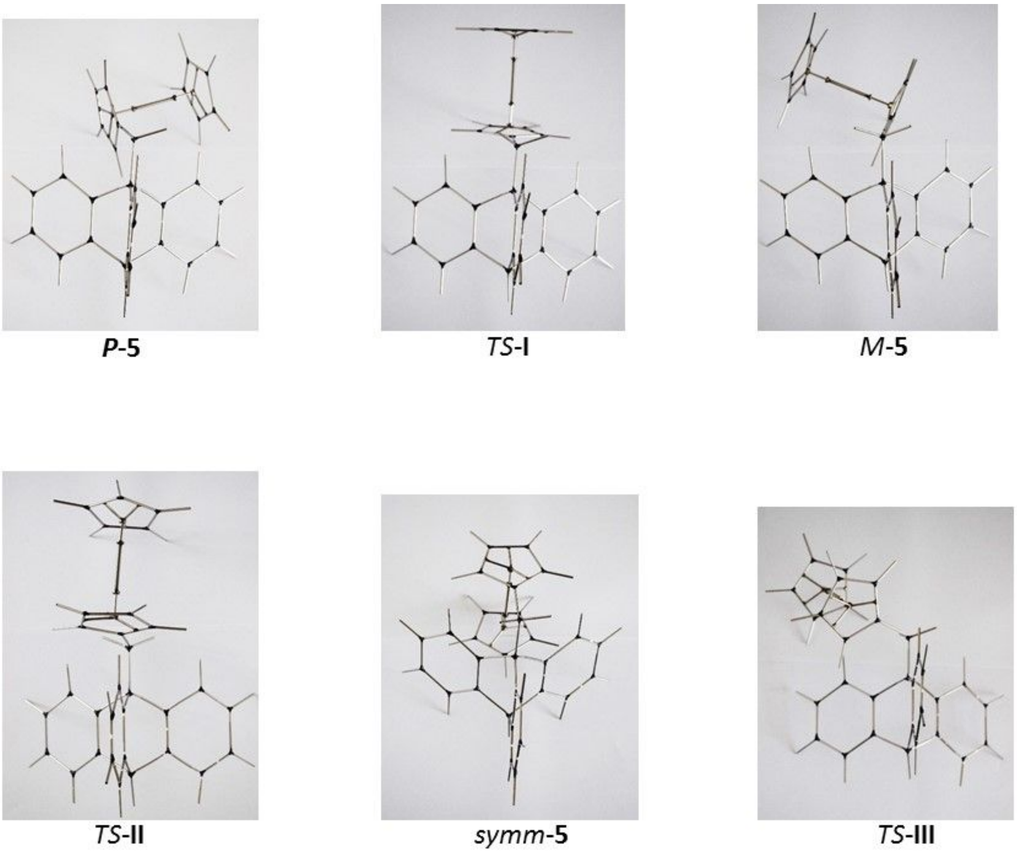

Figure S3. Photographic images of the molecular model of rotor **5**.

Movies S1, S2 and S3 illustrate, respectively, the calculated movements: oscillation, sliding and gearing type of the ferrocene fragment in **5**.

Movie TSI.

Animation of calculated molecular oscillation movement via TS-**I**.

Movie TSII.

Animation of calculated molecular sliding movement via TS-**II**.

Movie TSIII.

Animation of calculated molecular gearing movement via TS-**III**.
